# Experience of Bangladeshi Dental Students towards Online Learning during the COVID-19 Pandemic: A Web-Based Cross-Sectional Study

**DOI:** 10.3390/ijerph19137786

**Published:** 2022-06-24

**Authors:** Md Irteja Islam, Shah Saif Jahan, Mohammad Tawfique Hossain Chowdhury, Samia Naz Isha, Arup Kumar Saha, Sujan Kanti Nath, Mohammed Shahed Jahan, Md. Humayun Kabir, Ehsanul Hoque Apu, Russell Kabir, Nazeeba Siddika

**Affiliations:** 1 Sydney School of Public Health, Faculty of Medicine and Health, The University of Sydney, Sydney, NSW 2006, Australia; m.i.islam@sydney.edu.au; 2 Centre for Health Research and Faculty of Health, Engineering and Sciences, The University of Southern Queensland, Toowoomba, QLD 4350, Australia; 3 School of Allied Health, Faculty of Health, Education, Medicine and Social Care, Anglia Ruskin University, Chelmsford CM1 1SQ, UK; saifjahanddc@gmail.com (S.S.J.); russell.kabir@aru.ac.uk (R.K.); 4 Department of Dental Public Health, Sapporo Dental College, Dhaka 1230, Bangladesh; tawfique@sdch.edu.bd (M.T.H.C.); knsujan@yahoo.com (S.K.N.); 5 CAPABLE-A Cambridge-Led Programme in Bangladesh, University of Cambridge, Cambridge CB2 1TN, UK; samiaisha2@gmail.com; 6 Department of Dental Public Health, City Dental College, Dhaka 1229, Bangladesh; arupcdc@yahoo.com; 7 Department of Dental Public Health, Update Dental College, Dhaka 1711, Bangladesh; shahed.jahan5@gmail.com; 8 Department of Science of Dental Materials, Dhaka Dental College, Dhaka 1206, Bangladesh; drhumayunbulbul@gmail.com; 9 Department of Biomedical Engineering, Institute of Quantitative Health Science and Engineering, Michigan State University, East Lansing, MI 48824, USA; hoqueapu@msu.edu; 10 Division of Hematology and Oncology, Department of Internal Medicine, Michigan Medicine, University of Michigan, Ann Arbor, MI 48105, USA; 11 Department of Epidemiology and Biostatistics, College of Human Medicine, Michigan State University, East Lansing, MI 48824, USA

**Keywords:** COVID-19, online learning, dental students, LMIC, Bangladesh

## Abstract

Background: During the COVID-19 pandemic, dental education institutions throughout the world experienced significant challenges, including a quick shift to an online learning paradigm. Since the pandemic has had a considerable impact on dental education, this research evaluated the perspectives and attitudes towards online learning among undergraduate dental students in Bangladesh. Methods: The research was conducted through a cross-sectional method using self-administered online questionnaires. The questionnaire included information on the students’ sociodemographic status, their views, and their attitudes about the changes in the educational system, specifically regarding online learning. The study gathered data from 952 undergraduate students from 14 dental institutions in Bangladesh. Results: The results suggested that 87.5% of all the students were unsatisfied with their online programs. Most of the respondents who expressed dissatisfaction with their online classes were female, did not receive assistance in overcoming barriers to accessing the classes or materials (64.23%), did not have access to the institutional online-learning management system (OLMS) (67.35%), and did not have access to the online course materials (71.43%). The students considered that the shift to online learning failed to provide quality clinical teaching. Conclusions: The lack of an OLMS was found to be linked with dissatisfaction with online learning among Bangladeshi dental students during the COVID-19 outbreak. Additionally, insufficient time allocation and a lack of support through online training were found to be strongly correlated with the students’ discontent. The overall findings highlight the need to develop and implement effective online dentistry educational interventions to promote academic advancement and key practical skills.

## 1. Introduction

The highly infectious global pandemic, coronavirus disease 2019 (COVID-19), has had a devastating impact on health, economics, and social activities. It disrupted ways of life and prompted individuals to change their lifestyles, even in highly developed countries. Because of the severity of this pandemic, governments and institutions have taken drastic steps to ensure continuous learning [1]. Social distancing and avoiding large gatherings have become the norm, with traditional classrooms considered places in which the infection can spread [2]. Higher-education institutions worldwide unexpectedly switched from traditional learning systems to online learning to maintain the safety of students, teachers, and patients [1,2,3].

Online learning is a digital-technology teaching process facilitated remotely by internet from outside the classroom [4,5]. Globally, the concept of online learning is not new and has been used for decades in high-income countries [6]. In recent times, online learning has also progressed in low-and-middle-income countries (LMICs) due to the proliferation of high-tech devices and the availability of high-speed internet to young adults [6,7]. Online learning has become an internationally recognized way of providing learning environments that allow students to study in their own time, location, and schedule [8,9]. However, it has been reported that LMIC students lost nearly four months of education [10,11,12], compared with six weeks in high-income countries, during the COVID-19 pandemic [13].

Importantly, compared to other graduate or postgraduate teaching strategies, students pursuing courses and modules related to health and clinical education differ in learning their subjects via online education [14]. For instance, the dental curriculum is a blend of theoretical and practical learning. Online learning is generally only applicable to the theoretical content, while face-to-face interaction is significant for practical courses conducted in a one-to-one classroom setting. One challenge of online curricula is the lack of supervision and hands-on training. Health professionals’ education, including dental education, demands superior educational methods [2]. In addition, many students believe online exams lack fairness and prefer physical classroom examinations [15,16]. Even though online learning is widely accepted as a sound method, health-sciences students do not regard it as a replacement for in-person instruction, according to a study conducted in China [17]. A preliminary study aimed at exploring the initial reactions of European dental institutions revealed that most universities have started using online teaching strategies for non-clinical learning while enabling only limited clinical exposures [18]. However, there is limited scientific evidence on how online learning affects dental and medical education in LMICs, including its strengths and weaknesses [19,20].

Nevertheless, like many other LMICs, Bangladesh faces challenges in providing online teaching-and-learning facilities to tertiary students [21]. Despite the numerous challenges involved, the government of Bangladesh is committed to develop e-governance, e-learning, etc. [22]. While the progress on online education is visible from the Bangladesh government’s official website and posts, their impact on dental education is not identified. Even in traditional dental-education systems, we reported depression and hardship among the international students in Bangladesh studying dentistry [23]. In online learning environments, the students may require more time and interactive studies in order to understand topics. Dental education is still evolving, competitive and challenging, and dental colleges in Bangladesh have also incorporated new technologies and appropriate pedagogical tools in recent years. However, there are few published studies reporting on the aspects, such as curriculum design, training style, teaching strategies, and the overall educational environment, that affect the general preparedness of dental students. Further studies are required from different countries to obtain an overall perspective on the effectiveness of the online learning in dental education. The evaluation of the resources and quality of online learning and the assessment of student attitudes are essential to analyze the effectiveness of any online method of learning [24].

In a survey conducted during the COVID-19 pandemic in India, Pakistan, Nepal, Jordan, and Libya, most medical students negatively perceived or expressed dissatisfaction with online learning [25]. The pandemic has severely affected the self-confidence and performance levels of students studying dental courses globally [26]. In addition to medical students, dental students are considered among the group of students who are most exposed to various communicable diseases, including COVID-19. Dental students are also at increased risk of cross-infection because of their proximity with the oral cavities, the upper respiratory systems, saliva droplets, and blood of patients, as well as the usage of aerosol-generating instruments during clinical training [27,28,29]. Both dental students and educators have experienced challenges in pre-clinical and clinical dental education. These challenges have ranged from the fully online educational content to the limited opportunities for clinical dental training with patients for advanced final-year dental students [30]. One Serbian study reported that sudden and unintentional changes in teaching methods during lockdown and the restrictions imposed due to COVID-19 significantly hampered the self-confidence of final-year dental students [31]. During this period, Pakistani dental students were dissatisfied with the use of online learning platforms compared to one-to-one classrooms [32]. A study among Bangladeshi public and private university students from arts, science, and business backgrounds demonstrated a positive association between online learning and academic satisfaction [33]. However, to the best of our knowledge, no study has examined the views and/or experiences of Bangladeshi dental students of online learning during the COVID-19 period.

Therefore, in this study, we aimed to investigate dental students’ experiences and the factors associated with their levels of satisfaction with online learning in Bangladesh.

## 2. Materials and Methods

### 2.1. Study Design and Participants

An online cross-sectional study design was adopted. Being aged 18 years and above, enrolment as an undergraduate student at a dental college in Bangladesh, residency in Bangladesh during the COVID-19 pandemic period, and provision of electronic informed consent as a volunteer (without reward or incentives) were the eligibility criteria for this study.

### 2.2. Procedure

By convenience sampling, an online booklet regarding study background and self-administered online questionnaires (made by Google forms) with consent forms were distributed to the dental colleges in Bangladesh via email by the survey team to broaden the study’s scope. The online survey questionnaire was formulated based on a previous studies conducted in Pakistan [32] and Australia [34]; however, the survey team modified the questions according to the Bangladeshi context. The survey was divided into three sections. In the first section, the study participants were asked about their demographic details and year of study. In the second section of the survey, the students were asked about the quality of technological resources and general questions about online teaching. A five-point Likert scale was used in the third section of the survey to assess students’ perceptions of the effectiveness of online classes. The survey was conducted between 15 June 2021 and 1 July 2021. We collected data from a convenient sample from 14 dental institutions in Bangladesh. Participating dental colleges were: Dhaka Dental College, Sapporo Dental College, City Dental College, Rangpur Dental College, Update Dental College, Saphena Women’s Dental College, Sir Salimullah Medical College Dental Unit, Shaheed Suhrawardi Medical College Dental Unit, Mymensingh Medical College Dental Unit, Rajshahi Medical College Dental Unit, M.A.G. Osmani Medical College Dental Unit, Sher-E-Bangla Medical College Dental Unit, Marks Medical College Dental Unit, and Kumudini Women’s Medical College Dental Unit. Personal information (e.g., name, contact number, address) were not collected from the survey participants to ensure the privacy and reliability of data.

This study was conducted in accordance with the Declaration of Helsinki. Moreover, the Human Research Ethics Committee of the Sapporo Dental College and Hospital in Dhaka, Bangladesh approved this study (ref. no. SDC/C7/2021/810).

### 2.3. Measures

Dental students’ overall satisfaction with online classes during the COVID pandemic was selected as the outcome variable of the study. Students’ satisfaction was measured with the following question: ‘Overall, how satisfied were you with the online classes during COVID period compared to traditional active learning at campus?’. The answers were rated on a five-point Likert scale (very dissatisfied, dissatisfied, neither dissatisfied nor satisfied, satisfied, very satisfied). In this study, for the descriptive analyses, a new binary variable, ‘student satisfaction with online classes’, was created from the responses. Students who responded ‘satisfied’ or ‘very satisfied’ were classified as ‘satisfied’ (coded as 0); while those who answered, ‘very dissatisfied’, ‘dissatisfied’ or ‘neither dissatisfied nor satisfied’ were classified as ‘dissatisfied’ (coded as 1).

The following variables were used as potential covariates of interest in the study: gender (male, female), type of dental school (public, private), level of dental education (Year 1, Year 2, Year 3, Year 4), access to internet (easy, difficult), supply of electricity (uninterrupted, interrupted), type of electronic device used (smartphone, laptop/desktop/tab, combination), internet cost per month in Bangladeshi Taka (<500 BDT, 500–100 BDT, >1000 BDT), receipt of notification for online classes (on the same day, 1 day ago, ≥2 days ago), being assessed at the end of each online class (yes, no), availability of online learning management system (available, unavailable), availability of online lecture materials (available, unavailable), assistance in overcoming obstacles during/after online classes (yes, no), time allotted for online classes sufficient (yes, no), student–teacher interaction during/after online classes (interactive, not interactive).

### 2.4. Statistical Analysis

Initially, descriptive statistics in terms of frequency (n) and percentages (%), were calculated to describe the sample characteristics. Bivariate analyses using Pearson’s chi-square test were then conducted to predict the association between covariates and outcome variable (students’ overall satisfaction with online learning). Association yielding a *p*-value < 0.05 in bivariate analyses were subsequently included in the logistic regression models. A *p*-value at <0.05 was considered significant. The estimates of the logistic regression models were presented as both crude odds ratios (OR) and adjusted odds ratios (aOR) with their 95% confidence intervals (CI). The regression models were adjusted for each of the other variables simultaneously to derive the adjusted effect estimates. Further, to measure the overall performance of the used model, McFadden’s R^2^ [35], goodness-of-fit test [36] and link test [37] were performed. Additionally, the area under the ROC curve [38] were estimated to test predictive power of the model.

All analyses were carried out using Stata version 14.1 (Stata Corporation, College Station, TX, USA).

## 3. Results

A total of 952 participants completed the online questionnaire. Table 1 depicts their characteristics and online-learning platform-related background. Most of the participants were female (74.2%), and were studying in private dental colleges (76.5%) at the time the study was conducted. The sample had a fair representation of all years of dental school, i.e., first year (31.9%), second year (21.3%), third year (25.3%), and fourth year (21.4%). Regarding the online-learning platforms, most of the participants had easy access to the internet (79.6%), around 95% of the sample used smartphones, and almost 54% of the participants reported interrupted electricity supply. The majority (86.9%) of the students reported having used their own devices for the online classes. Of all the students who participated, 87.5% reported dissatisfaction with online classes.

**Figure 1:**
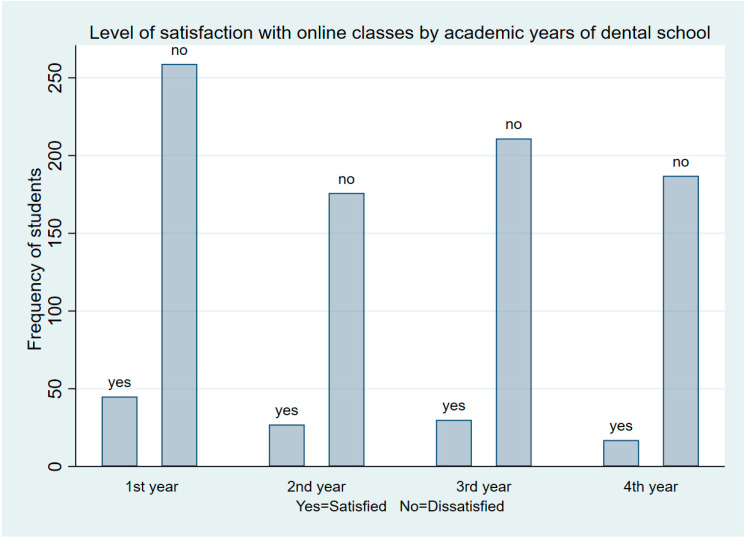
Satisfaction levels of students with online classes by academic years of dental school.

Figure 1 illustrates that the dissatisfaction level was higher among first-year students, followed by third, fourth, and second-year students.

Table 2 presents the results from the bivariate analysis on the association between the levels of satisfaction and the factors related to online classes. The factors that showed statistically significant associations (*p* < 0.05) with the levels of satisfaction were “gender”, “being assessed at the end of each class”, “availability of OLMS in the institutions”, “availability of the online course materials”, “assistance in overcoming obstacles in accessing the classes or materials”, “time allotted for online classes is sufficient”, and “student–teacher interaction during or after the class”. Among the female respondents, 89.24% reported dissatisfaction, whereas 82.52% of the males were dissatisfied with the online classes. Having interrupted electricity was more closely associated with higher dissatisfaction (88.85%) than uninterrupted electricity (85.94%). Furthermore, the students using smartphones showed higher dissatisfaction (87.80%) than the students using laptops/desktops/tablets (79.49%). A greater number of students (92.56%) showed dissatisfaction who were not helped to overcome the obstacles to accessing the classes or materials compared to the students who were assisted (79.68%). Of the students for whom the institutional OLMS was unavailable, 91.67% showed dissatisfaction, whereas 80.00% of those who had institutional OLMS were dissatisfied. Similarly, a greater number of the students for whom the online course materials were unavailable reported dissatisfaction (90.84%), whereas 80.13% of the students who had access to online course materials were dissatisfied.

The factors related to dissatisfaction with online classes are shown in Table 3. The odds of dissatisfaction were 1.82 (aOR:1.82; 95% CI:1.19, 2.80) times higher among female students than males. The students who reported that their institution did not have OLMS were 1.97 times (aOR: 1.97; 95% CI: 1.14, 3.41) more dissatisfied than those who had institutional OLMS. Similarly, the students who were not assisted in overcoming the obstacles to access to classes or materials, who reported insufficient time allotment for online classes, and who could not interact with their teachers (during and after class) showed 1.78 times (aOR: 1.78; 95% CI: 1.12, 2.80), 2.11 times (aOR: 2.11; 95% CI: 1.28, 3.46), and 1.62 times (aOR: 1.62; 95% CI: 1.02, 2.59) higher odds of dissatisfaction, respectively, compared to those who reported otherwise. The McFadden’s R^2^ was 0.074, the goodness-of-fit test was not significant (*p* > 0.05), and the link test statistics were statistically significant (*p* < 0.001), showing that the regression model fits reasonably well. Moreover, the area under the ROC curve was 0.72, indicating the good predictive power of the fitted model.

## 4. Discussion

The COVID-19 pandemic has resulted in a disruption of education systems on an unprecedented scale, and dental education is no exception to this [39,40]. In response to the pandemic situation around the globe, face-to-face learning has been disturbed and online learning platforms have taken on a significant role in dental education [2,39,41]. However, information on dental students’ experiences of online dental education during the COVID-19 pandemic is limited [42], particularly in lower-and-middle income countries, such as Bangladesh. This study evaluated the overall levels of satisfaction and identified factors associated with online learning among undergraduate dental students of Bangladesh during the COVID-19 pandemic.

We found that most of the dental students were not satisfied with online learning and preferred campus-based courses to online education, which is corroborated by previous research [1,42,43]. Since dental education is composed of several components, such as lectures/tutorials, problem-based learning interactions, research-based learning, simulated training courses, and clinical skills training [42,43], studies reported that online classes are not feasible for professional dental courses [42,44]. Other studies suggested that blended learning may be useful for dental education, with lectures/tutorials carried out remotely and clinical/professional training courses arranged on-site [41,44]. Moreover, dissatisfaction may arise from the fact that both students and teachers needed to become familiar with online learning [45].

Our study also revealed that female dental students were more likely to be dissatisfied with online learning compared to their counterparts. A possible explanation could be that males generally have more interest and knowledge in technological innovations, whereas females report experiencing more difficulties and lower levels of interest [46,47].

In addition, the results suggested that the unavailability of an online-learning management system (OLMS) was significantly associated with dissatisfaction with online learning among Bangladeshi dental students during the COVID-19 pandemic. Since the shift from on-site classes to online learning was unanticipated and needed swift implementation for education to continue, the rapid development of an efficient OLMS was a challenge during the pandemic [40,48,49,50,51]. Previous studies reported that an established online educational system can help dental students to improve their theoretical as well as pre-clinical/clinical skills [44,49,50].

Moreover, inadequate time allocation and a lack of assistance regarding online classes were also found to be significantly associated with dissatisfaction. This may have been because online learning was new to both the dental students and their teachers [39,50]. Evidence suggests that both students and teachers encountered multiple problems (e.g., failure to adapt to technology, poor time management, lack of communication, insufficient support services) due to the sudden and complete changeover during COVID-19, which subsequently resulted in dissatisfaction with online learning [45,47,48]. Furthermore, we found that a lack of interaction between students and teachers was more likely to increase dissatisfaction with online classes, which is consistent with past research findings [33,44,46]. This may have been due to that fact that the nature of student–teacher interactions suddenly changed during the pandemic [52]. A lack of practical online-education training was reported to be a significant problem, contributing to poor student–teacher interactions [53,54].

Because of the COVID-19 pandemic, many students felt that switching to online platforms hampered their ability to acquire clinical skills and professionalism compared to what they had experienced in conventional classroom settings. When the practical hands-on assessment was cancelled, third- and final-year professional examinees lost almost four months of clinical practice. The participants in the survey felt they had lost out on significant learning opportunities, especially in clinical settings. The study indicated that the impact of the COVID-19 pandemic on the clinical abilities of the final-year students who did not have enough time to compensate for their missed educational time is challenging to quantify, and the ramifications may not be apparent for many years. A long break from clinical training might affect dental students’ confidence and learning ability.

It was also noticed that the students from Year 1 were the most dissatisfied. Most of the dental students came different parts of the country and were admitted on the assumption of an in-person learning environment; hence, they lacked technological knowledge, especially of the kind required for OLMS and online-learning environments. As a result, it was difficult for them to become proficient with virtual learning methods within the short period of time spanning the COVID lockdown phases. Most residential hostels and dormitories were closed, as were commercial complexes, which made it difficult for the students and teachers to equip themselves with modern technical appliances. Moreover, first year-dental students face the challenges and new experiences associated with greater workloads than they are used to. They require more support and face-to-face interaction to build up their understanding. This might be a reason behind their dissatisfaction with online learning. Furthermore, during the data collection period, the uncertain future of the learning methods, in-person classes, and unexpected delays to the examination schedules might have created fear and depression among the students. This situation might have caused their opinions to become more unfavorable compared to the other students (second year and onwards), who were more advanced and accustomed to the learning system.

Considering the ongoing COVID-19 pandemic and future public-health disasters, technological advancements, such as online learning tools, live lecture streaming, high-speed internet, and blended interactive educational approaches will continue to influence education systems and guide the preparations of dental education institutions. When the pandemic began, we noticed a novel way to ease the transition into clinical experience and created adjusted or innovative ways to ensure that students obtain the clinical experience they require. Our research found that virtual education has a negative impact on educational quality. Thus, we recommend the optimization of online learning techniques for efficiently delivering high-quality dental education. For instance, governments, healthcare authorities, and dental schools need to adapt their education systems to improve the one-to-one, didactic, preclinical, clinical, administrative, and research components of dental education and implement effective online-learning education systems in response to the changes caused by COVID-19 [26]. Dental institutions should provide students with internet wi-fi devices and affordable purchasing options for portable devices (e.g., laptops, notebooks, and tablets). It can be conclusively stated that the current pandemic has irrefutably exposed the limitations and barriers associated with the adaptation of online teaching resources in dental colleges in Bangladesh.

### Strengths and Limitations

To the best of our knowledge, this is the first study to assess the impact of the COVID-19 pandemic on online learning among undergraduate dental students in Bangladesh. Furthermore, this study features a reasonable sample, with students from 14 dental medical schools in Bangladesh, across all pre-clinical and clinical years. However, our study has some limitations. First, our study lacks temporal causality due to its cross-sectional design. To be precise, longitudinal studies using a detailed face-to-face questionnaire along with a qualitative study should be carried out to assess and identify the factors affecting the levels of satisfaction with online learning among dental and/or medical students. Second, since our study respondents were required to have access to the internet to complete the online survey, and we collected data from a convenient sample of 952 students from 14 dental institutions out of a total of 28 listed dental institutions, according to the Bangladesh Medical and Dental Council (BM&DC) website (https://www.bmdc.org.bd/about-college-n; accessed on 19 June 2022), this may have limited the representativeness of our study population. Additionally, self-reporting regarding satisfaction/dissatisfaction about online learning, monthly internet costs, time allocation for online classes, and interactions with teachers may result in recall bias and/or social-desirability bias.

## 5. Conclusions

Among dental students, face-to-face education remains the preferred method of learning. Dental schools should explore and standardize the use of online teaching techniques to theoretically educate their students and provide basic practical information about the numerous processes that they will experience when the schools open. Time and experience will be necessary to transition from conventional face-to-face teaching to a fully operational virtual educational system. To maintain and continually enhance the quality of their online course materials, dental colleges need to invest significantly in faculty professional development. However, schools must also be aware of the potential gaps and issues connected with such teaching-and-learning approaches and endeavor to minimize them whenever feasible. The findings of this and related research may be utilized to modify the current online learning paradigm to better meet the requirements of students. The findings of this study will help to create a better understanding of the challenges associated with online learning that students experience. As a result, dental schools may use this information to develop an online teaching platform that focuses on enhancing the educational experience of students.

## Figures and Tables

**Table 1:** Characteristics of the study population.

Characteristics	*n* (%)
Total	952 (100)
Age (Mean ± SD)	21.7 (1.66)
Gender	
Male	246 (25.8)
Female	706 (74.2)
Type of institute	
Private	728 (76.5)
Public	224 (23.5)
Year of dental school	
1st	304 (31.9)
2nd	203 (21.3)
3rd	241 (25.3)
4th	204 (21.4)
Access to internet	
Easy	758 (79.6)
Difficult	194 (20.4)
Supply of electricity	
Interrupted	511 (53.7)
Uninterrupted	441 (46.3)
Type of electronic device used	
Smartphone	910 (95.6)
Laptop/desktop/tablet	39 (4.1)
Combination	3 (0.3)
Device owned	
Student	828 (86.9)
Others	125 (13.1)
Level of satisfaction	
Satisfied	119 (12.5)
Dissatisfied	833 (87.5)

**Table 2:** Bivariate analysis (Pearson’s chi-square) between several factors and level of satisfaction with online classes.

Variables	Satisfied *n* (%)	Dissatisfied *n* (%)	*χ* ^2^	*p*-Value
Gender				
Male	43 (17.48)	203 (82.52)	7.52	0.006
Female	76 (10.76)	630 (89.24)		
Type of Institute				
Private	94 (12.91)	634 (87.09)	0.48	0.488
Public	25 (11.16)	199 (88.84)		
Year of dental school				
1st	45 (14.80)	259 (85.20)	4.83	0.185
2nd	27 (13.30)	176 (86.70)		
3rd	30 (12.45)	211 (87.55)		
4th	17 (8.33)	187 (91.67)		
Access to internet				
Easy	92 (12.14)	666 (87.86)	0.44	0.503
Difficult	27 (13.92)	167 (86.08)		
Supply of electricity				
Interrupted	57 (11.15)	454 (88.85)	1.83	0.177
Uninterrupted	62 (14.06)	379 (85.94)		
Type of electronic device used				
Smartphone	111 (12.20)	799 (87.80)	2.79	0.247
Laptop/desktop/tab	8 (20.51)	31 (79.49)		
Combination	0 (0.00)	3 (100.00)		
Monthly Internet cost (in BDT)				
Less than 500	31 (13.14)	205 (86.86)	0.30	0.860
500–1000	67 (12.01)	491 (87.99)		
More than 1000	21 (13.29)	137 (86.71)		
Notification of online class schedule received				
On the same day	35 (14.40)	208 (85.60)	1.99	0.370
1 day before	67 (12.52)	468 (87.48)		
2 or more days before	17 (9.77)	157 (90.23)		
Being assessed at the end of each class				
Yes	79 (14.82)	454 (85.18)	5.97	0.015
No	40 (9.55)	379 (90.45)		
Availability of online LMS or Institutional Website				
Available	68 (20.00)	272 (80.00)	27.20	0.000
Unavailable	51 (8.33)	561 (91.67)		
All key information about the course is available on learning management system				
Available	59 (19.87)	238 (80.13)	21.41	0.000
Unavailable	60 (9.16)	595(90.84)		
Students are assisted in overcoming obstacles in accessing the classes or materials?				
Yes	76 (20.32)	298 (79.68)	34.45	0.000
No	43 (7.44)	535 (92.56)		
Time allotted for online classes is sufficient?				
Yes	93 (17.78)	430 (82.22)	29.61	0.000
No	26 (6.06)	403 (93.94)		
Student–teacher interaction during or after classes				
Interactive	85 (17.75)	394 (82.25)	24.25	0.000
Not interactive	34 (7.19)	439 (92.81)		

Note: OLMS, online-learning management system, *χ*^2^, Chi-square.

**Table 3:** The factors associated with dissatisfaction with online classes among the overall study population.

Factors	Crude OR (95% CI)	Adjusted OR (95% CI)
Gender		
Male	Ref.	Ref.
Female	1.76 (1.17, 2.64)	1.82 (1.19, 2.80)
Assessment at the end of each class		
Yes	Ref.	Ref.
No	1.65 (1.10, 2.47)	1.08 (0.69, 1.67)
OLMS (availability of online LMS or institutional website)		
Yes	Ref.	Ref.
No	2.75 (1.86, 4.07)	1.97 (1.14, 3.41)
All key information about the course is available in OLMS		
Yes	Ref.	Ref.
No	2.46 (1.67, 3.63)	0.94 (0.53, 1.65)
Assistance with overcoming obstacles to accessing classes or materials		
Yes	Ref.	Ref.
No	3.17 (2.13, 4.73)	1.78 (1.12, 2.80)
Time allotted for online classes sufficient?		
Yes	Ref.	Ref.
No	3.35 (2.13, 5.29)	2.11 (1.28, 3.46)
Interaction with teacher (during + after class)		
Yes	Ref.	Ref.
No	2.79 (1.83, 4.24)	1.62 (1.02, 2.59)
Model performance test		
McFadden’s R^2^: 0.074		
Goodness-of-fit test statistic (*p*-value): 139.31 (0.97)		
Link test: 1.94 ***		
Area under the ROC curve: 0.7241		

Note: Estimates are derived from the logistic regression model. Adjusted effect estimates are adjusted for each of the other variables simultaneously. OR, odds ratio; OLMS, online learning management system. *** (*p* < 0.001).

## Data Availability

The data presented in this study are available on reasonable request from the corresponding author.
